# Contact allergy to an electronic device

**DOI:** 10.1111/ddg.15775

**Published:** 2025-05-13

**Authors:** Margitta Worm, Julia Oberschmied, Elsbeth Oestmann

**Affiliations:** ^1^ Department of Dermatology Venereology and Allergy Charité – Universitätsmedizin Berlin Berlin Germany

Dear Editors,

Apple products are used commonly worldwide including earphones, smart phones, watches and other electronic devices. Earphones and watches pose a risk of contact sensitization due to the direct skin contact of the devices. Although these are frequently used in the general population, only a few cases of allergic contact dermatitis due to such products have been reported.^1^, ^2^, ^3^ Acrylates have been implicated as the causing contact allergens by patch testing. Earphone devices from Apple^®^ such as AirPods^®^ may contain trace amounts of acrylates and methacrylates from adhesives.^4^

We report on a 53‐year‐old male patient who presented with eczematous skin lesions on the left dorsal wrist, localized to the contact area of his Apple Watch^®^ (Figure 1). He also reported eczematous skin reactions in the external auditory meatus after using his Apple AirPods^®^ in‐ear headphones. No allergies were known, but chronic coughing and asthma were reported. The patient had cats at home. Regular medication was denied. Oral mucosal symptoms had occurred after a dental procedure with filling material of unknown origin several years ago.

**FIGURE 1:**
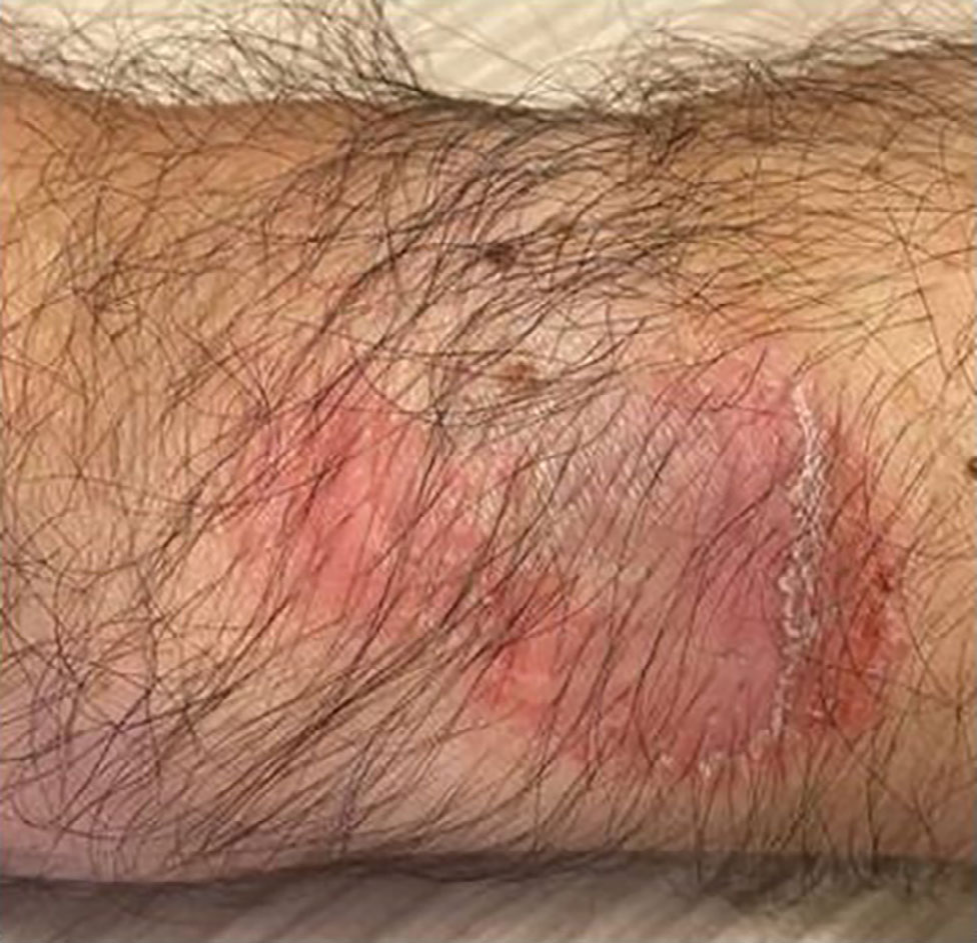
Eczematous skin lesion in the contact area of the watch.

The patch test with the standard test battery and the dental technician test battery of the German contact allergy group (DKG) revealed twofold positive (++) reactions to hydroxyethyl acrylate, 2‐hydroxypropyl methacrylate, and isobornyl acrylate, and positive reactions (+) to 2‐hydroxyethyl methacrylate, ethylene glycol dimethacrylate, and triethylene glycol dimethacrylate after 72 hours (Figure 2). The skin prick test for the most frequent inhalant allergens was positive for house dust mites. Total IgE was 24 KU/l. No specific IgE against cats and very low specific IgE against the house dust mite were found. A differential blood count, a lung function test and the exhaled nitric oxide test (NO) were normal.

**FIGURE 2:**
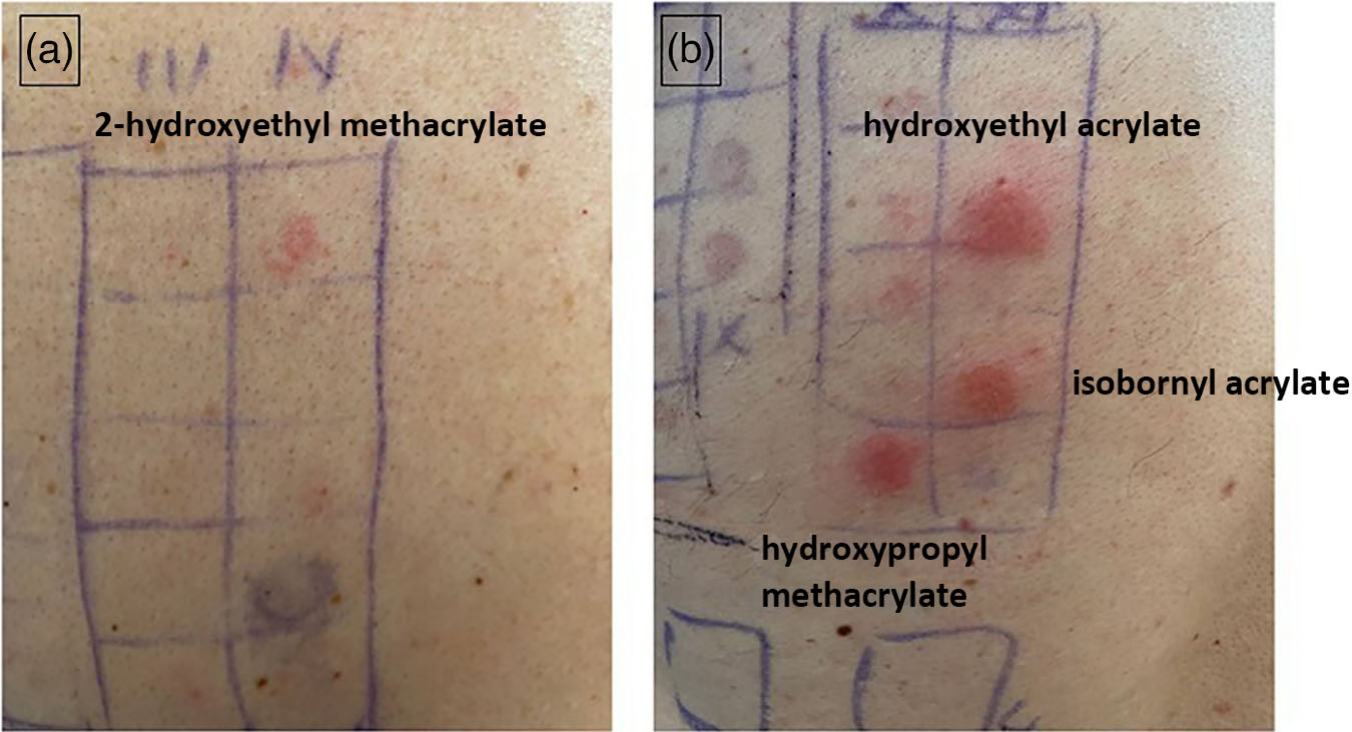
(a, b) Positive patch test reactions against acrylates.

We identified acrylates and methacrylates as the source of the eczematous skin lesions in the external auditory meatus and on the wrist due to exposure to the products Air Pods^®^ and Apple Watch^®^.

Electronic devices which are in permanent close contact with the skin may cause contact sensitization due to their acrylate content. It is well known that acrylate monomers are potent sensitizers,^2^, ^4^ while the risk of developing an allergy to polymerized acrylates and methacrylates is low. However, incomplete polymerization may leave trace amounts of monomers.^2^ Interestingly we also observed a clear reactivity towards isobornyl acrylate and 2‐hydroxypropyl acrylate in our patient. Isobornyl acrylate has been reported as a potent sensitizer in continuous glucose monitoring devices in patients with type 1 diabetes.^5^, ^6^ Sensitization against isobornyl acrylate in glucose monitoring systems has been suspected to develop from diffusion of the allergen from the glue used in the assembly of the device.^2^ Moreover isobornyl acrylate has also been described as an occupational contact allergen present in cell phone screen protectors.^7^

In patients with contact allergy to Air Pods^®^ a co‐sensitization to isobornyl acrylate and 2‐hydroxypropyl acrylate with methyl acrylate sensitization has also been described.^2^

We suspect that the sensitization in our patient occurred during a dental procedure with acrylate containing filling material. Exposure to unpolymerized (meth)acrylates is a relevant cause of occupational allergic contact dermatitis in dental technicians and dentists.^8^ The clinical reactions, but possibly also sensitization related to the AirPod^®^ and the Apple Watch^®^ themselves may have resulted from the intense exposure. Today, various acrylates and methacrylates are contained in medical products, e.g. orthopedic materials, surgical glues, and adhesive tapes. (Meth)acrylates are also frequently present in nail products, posing a growing health problem for consumers and professionals. They have recently been declared emerging contact allergens.^4^, ^9^ The lack of ingredient labelling in medical devices in particular remains an obstacle to the investigation of allergic reactions. Currently, the broad occupational and health related aspects of acrylate allergy, coupled with a rapidly increasing incidence, are fueling the discussion on how the risk for consumers and patients can be mitigated.

## CONFLICT OF INTEREST STATEMENT

None.
